# Supplementary material for: Collaborative intelligence in AI: Evaluating the performance of a council of AIs on the USMLE
Source: PLOS Digit Health. 2025 Oct 9;4(10):e0000787. doi: 10.1371/journal.pdig.0000787 (PMC12510544; doi:10.1371/journal.pdig.0000787)
Supplement: S2 File — (ZIP) [file pdig.0000787.s002.zip › S2 Data Raw data files containing unprocessed question inputs and LLM outputs including transcripts of deliberations/STEP3_Question-124-1_Type-MC-NJ.docx]

**Facilitator** **[Round 1]:** A 78-year-old man, who underwent coronary angiography with stent placement 8 years ago, comes to the office for a routine health maintenance examination. The patient reports no new symptoms except for shortness of breath and fatigue during exercise that began 3 months ago when he tried to increase the distance of his daily walk from 1 mile to 3 miles. He has not had orthopnea, paroxysmal nocturnal dyspnea, or chest pain. Additional medical history is significant for hypertension, coronary artery disease, dyslipidemia, and polymyalgia rheumatica. The patient also has a heart murmur that was discovered at an armed forces screening physical examination at age 18 years, but it did not prevent him from serving in the military. Medications are metoprolol, atorvastatin, lisinopril, omeprazole, and a low dose of prednisone every other day. The patient is active and manages a small farm. BMI is 26 kg/m². Vital signs are temperature 37.0°C (98.6°F), pulse 60/min, respirations 18/min, and blood pressure 124/61 mm Hg. Cardiac examination discloses a normal S1, pronounced S2, and a grade 2/6 late systolic murmur heard at the right upper parasternal border. Pulses are normal in contour. Results of laboratory studies are within the reference ranges. ECG shows no abnormalities. Which of the following is the most appropriate next step in management? (A) Decrease the dose of metoprolol (B) Increase the dose of prednisone (C) Obtain serum vitamin D concentration (D) Order a transthoracic echocardiography

**AI_D:** (1) RESTATING THE QUESTION: The question is asking what the most appropriate next step would be in managing the medical care of a 78-year-old man who has a history of coronary heart disease, was found to have stent placement 8 years ago, and is now experiencing shortness of breath and fatigue during exercise. The symptoms started 3 months prior when he tried to increase his daily walking distance. (2) PLANNING: This patient's presentation can suggest multiple possibilities. The onset of shortness of breath and fatigue with exercise could suggest worsening of his cardiovascular disease, which could mean issues with his stent placement. Or it could suggest something related to his current medications, like metoprolol or prednisone, which could be leading to symptoms. Alternatively, it could be due to another unrelated condition such as Vitamin D deficiency or even an unknown condition related to his heart murmur. All these assumptions could potentially explain his symptoms, but it's important to clinically prioritize which should be pursued. (3) CLINICAL REASONING: Given the history of coronary artery disease and his symptoms of shortness of breath and fatigue during exercise, it would make sense to first investigate any potential cardiovascular cause. His cardiac examination reveals a late systolic murmur at the right upper parasternal border, which is suggestive of aortic stenosis. This murmur along with his current symptoms could suggest an undiagnosed or progressive aortic stenosis, which won't be apparent with electrocardiogram only. (4) CONCLUDING: Based on my clinical reasoning, acquiring more diagnostic information regarding the condition of his heart seems to be a high priority. Testing with echocardiography would provide a detailed view of the patient's heart valves and could help detect, verify, or rule out aortic stenosis or other heart conditions that could potentially be causing his symptoms. (5) RESPONSE: Therefore, to better understand the cause of this patient's symptoms and to confirm or rule out potential cardiovascular causes, the most appropriate next step in management would be to (D) Order a transthoracic echocardiography.

**AI_C:** RESTATING THE QUESTION: The case presents a 78-year-old male with a notable medical history of coronary angiography with stent placement, hypertension, coronary artery disease, dyslipidemia, and polymyalgia rheumatica. He reports increased shortness of breath and fatigue during exercise, with no other acute symptoms. A previously identified heart murmur is noted on a recent cardiac examination. His current medications include metoprolol, atorvastatin, lisinopril, omeprazole, and prednisone. The question now is which management step should be prioritized in his case? PLANNING: The alternate assumptions to be generated are based on the four options. Either (A) his medication dosage needs adjustment, specifically metoprolol, or (B) his prednisone dosage needs to be increased or (C) his serum vitamin D concentration needs to be evaluated or (D) a transthoracic echocardiography needs to be ordered. CLINICAL REASONING: Considering the patient's medical history, his current symptoms of shortness of breath and fatigue during exercise, and findings from the cardiac examination, it seems most relevant to investigate a possible cardiac origin for these new developments. His medications, notably metoprolol and prednisone, have certain side effects, but there are no immediate indications that these medications need dosage adjustments. The patient's serum vitamin D concentration could be of interest, but his symptoms do not specifically suggest a vitamin D deficiency. Hence, the assumption leading to a decision to order a transthoracic echocardiography (option D) appears more plausible. CONCLUDING: Ordering a transthoracic echocardiography would help evaluate the structural and functional aspects of the patient's heart, potentially revealing if his heart murmur or any other cardiac abnormalities are contributing to his recent fatigue and shortness of breath during exercise. RESPONSE: Therefore, the most appropriate next step in management is to order a transthoracic echocardiography (D).

**AI_A:** (1) RESTATING THE QUESTION: This question is asking about the most suitable next step in managing a 78-year-old man with a significant cardiac history, current heart murmur, and recent symptoms of fatigue and shortness of breath during exercise. These symptoms started when he tried to increase his daily walk. He has no orthopnea or chest pain. His medications include beta-blocker metoprolol, statin atorvastatin, ACE inhibitor lisinopril, proton pump inhibitor omeprazole, and corticosteroid prednisone. His physical examination shows a normal S1, pronounced S2, and a grade 2/6 late systolic murmur. His ECG and lab results are normal. (2) PLANNING: The primary assumption is that the man's symptoms of fatigue and shortness of breath are related to his cardiac conditions, compounded by his increased physical activity recently. However, this assumption could be challenged with the question: could his symptoms be caused or exacerbated by something else apart from the cardiac issues? (3) CLINICAL REASONING: The patient has a significant cardiac history and is on medication for this, including metoprolol. However, his new symptoms of fatigue and shortness of breath occurring specifically during increased exercise could potentially indicate a functional or structural issue with his heart that is becoming more evident with increased physical demand. (4) CONCLUDING: His presenting symptoms, along with his cardiac history and murmur but lack of abnormalities on ECG or lab tests, suggest the need for further diagnostic imaging to better understand the structure and function of his heart. (5) RESPONSE: Therefore, the most appropriate next step in management for this patient would be (D) to order a transthoracic echocardiography. This would allow for visualization of the heart chambers, valves, and the ability to measure ejection fraction and cardiac output, potentially explaining the patient's symptoms during exercise.

**AI_B:** RESTATING THE QUESTION: The query involves a 78-year-old man with a clinical history of coronary artery disease, hypertension, and dyslipidemia. He had a coronary angiography and stent placement 8 years ago. He is evidently health-conscious, increasing his daily walking distance, however, this has led to fatigue and shortness of breath, which is new for him. He has a heart murmur - discovered when he was 18 years old. His medications include metoprolol, atorvastatin, lisinopril, omeprazole, and occasional prednisone. His physical exam is mostly normal with an exception of a late systolic murmur. The question is: of the listed options (decreasing metoprolol dose, increasing prednisone dose, obtaining serum vitamin D concentration, ordering a transthoracic echocardiography), what would the best next step in management be? PLANNING: The assumptions here could be that the patient's symptoms are due to his recent increase in physical activity, or perhaps that they are an indication of progression or complications related to his known medical conditions. These are rather significant assumptions that need adequate investigative approaches. By using a spectrum of medical knowledge, it might be beneficial to rule out cardiac issues due to the patient's history of heart disease. A transthoracic echocardiogram is a noninvasive diagnostic tool that could provide valuable information in this situation. CLINICAL REASONING: The patient's symptoms of fatigue and shortness of breath during exercise may be related to his heart condition. The presence of a heart murmur further boosts this relevance as it often indicates a structural heart disease. Ordering a transthoracic echocardiography will allow the healthcare provider to visually examine the size, shape, and function of the heart directly and assess for any structural abnormalities that could be contributing to the patient's symptoms. This practice matches with the steps in evidence-based management of cardiac symptoms, which prioritize examination and diagnosis of the cardiac function and structure. CONCLUDING: By connecting his medical history, current symptoms, and presence of a heart murmur, it is fair to hypothesize that the patient might have developed some changes in his cardiac status, which could explain his fatigue and shortness of breath. Altering medication doses or sending for a vitamin D level test without assessing for potential cardiac changes first might not address the root cause of his symptoms. RESPONSE: Therefore, based on the information available and guided by evidence-based practice, the most appropriate next step in managing this patient would be to (D) order a transthoracic echocardiography.

**AI_E:** This query focuses on the most appropriate next action in the treatment of a 78-year-old man. He underwent stent placement following a coronary angiography 8 years ago and is now experiencing shortness of breath and tiredness during exercise, which began when he started to extend his daily walk from one to three miles. This patient has no other new symptoms, such as paroxysmal nocturnal dyspnea, chest pain, or orthopnea. He has a substantial medical history, which includes dyslipidemia, hypertension, coronary artery disease, and polymyalgia rheumatica. From the time he undertook a physical examination during his armed services screening at 18, he's had a heart murmur. His medication regime includes metoprolol, atorvastatin, lisinopril, omeprazole, and prednisone in low doses every alternate day. A late systolic murmur, grade 2/6, is evident in his cardiac examination, along with a pronounced S2 and normal S1. Current analysis and ECG reveal no irregularities. Hence, the question seeks to investigate the next suitable step needed for managing this case. A lot of assumptions can be implied. For instance, his symptoms might be suggestive of a heart failure or a functional decline due to old age or, because he's on low doses of prednisone every other day, it might be related to his polymyalgia rheumatica or possible side effects of the drug. It's also possible that the late systolic murmur heard could indicate an underlying valvular heart disease, considering his age and history. However, his age, history, lack of symptoms suggestive of heart failure (such as orthopnea, paroxysmal nocturnal dyspnea, or chest pain), presence of a heart murmur since young age and new onset exercise intolerance prioritize the assumption that a structural heart disease might be responsible for these symptoms. Upon further clinical evaluation, it's clear that although his symptoms could be attributed to his polymyalgia rheumatica and the associated prednisone use, a more sinister cause may exist. While an age-related functional decline can cause such symptoms as fatigue and shortness of breath, the presence of a heart murmur in this patient raises the possibility of a structure heart disease, particularly valvular, given his long-standing murmur, history of heart disease and the absence of heart failure symptoms. In step-wise reasoning, we can look into the possibility of decreasing his medication doses, particularly metoprolol or increasing the dose of prednisone to manage his polymyalgia rheumatica in attempt to alleviate the symptoms. However, these options were disregarded due to lack of evidence suggesting a flare up of his rheumatica condition or his medical condition requiring an adjustment in his beta blocker therapy. Vitamin D concentration is also an option, but there is no indication or symptoms suggesting deficiency in this patient. The likely best approach appears to be to delineate his heart structure and function, to ascertain whether there has been developments in his status quo. In summary, the most clinically priority in this patient is to evaluate his heart status, particularly the valve function, given his history of murmur, coronary artery disease, absence of heart failure symptoms and new onset exercise intolerance. So, an echocardiography would help to better elucidate his heart structure and function in the setting of his new onset symptoms. So, (D) Order a transthoracic echocardiography would be the most appropriate step in managing this patient currently.

**Facilitator** The consensus among the AI responses is that the most appropriate next step in managing the 78-year-old man's case would be to order a transthoracic echocardiography (Option D). This decision is based on the patient's significant cardiac history, his recent symptoms of fatigue and shortness of breath during increased physical activity, and the presence of a heart murmur. Despite the lack of abnormalities on his ECG or lab tests, these factors suggest a potential functional or structural issue with his heart that is becoming more evident with increased physical demand. A transthoracic echocardiography would allow for visualization of the heart chambers, valves, and the ability to measure ejection fraction and cardiac output, potentially explaining the patient's symptoms during exercise. This approach aligns with evidence-based management of cardiac symptoms, which prioritize examination and diagnosis of the cardiac function and structure. Therefore, before considering adjustments to his medication or investigating other potential causes such as vitamin D deficiency, it is crucial to first assess for potential cardiac changes that might be the root cause of his symptoms.
